# Pulmonary Embolism: Webcast June 13 2024

**DOI:** 10.14797/mdcvj.1526

**Published:** 2024-12-26

**Authors:** Alan B. Lumsden, Thomas M. Loh, Patrick E. Muck, Pavan Thangudu, Joseph J. Naoum, Nicolas J. Mouawad, Paul Haddad, Zachary Steinberg

**Affiliations:** 1 Houston Methodist DeBakey Heart & Vascular Center, Houston Methodist, Houston, Texas, US; 2 Houston Methodist, The Woodlands, Texas, US; 3 Good Samaritan Hospital, Cincinnati, Ohio, US; 4 Bethesda North Hospital, Cincinnati, Ohio, US; 5 Pulmonary Disease & Critical Care, Memorial Hermann Health System, The Woodlands, Texas, US; 6 Houston Methodist Hospital Clear Lake, Nassau Bay, Texas, US; 7 Houston Methodist DeBakey Heart & Vascular Center, Houston, Texas, US; 8 McLaren Health System, Bay City, Michigan, US; 9 Houston Methodist Hospital, Houston, Texas, US; 10 University of Washington, Seattle, Washington, US

**Keywords:** pulmonary embolism, pulmonary embolism response team (PERT), massive PE, deep vein thrombosis (DVT), venous thromboembolism (VTE), chronic thromboembolic pulmonary hypertension

## Abstract

This 61-minute webcast features a conversation about “Pulmonary Embolism”—the focus of Issue 20.3. Hosted by the issue’s editors, the discussion engages the authors on emerging themes and lessons learned while researching and writing the articles. View the video at https://livestream.com/accounts/21157318/events/11146629.

## Introduction

This 61-minute webcast (Video 1) features the issue’s guest editors conversing with four of the issue’s authors on the emerging themes and lessons learned while exploring the article topics related to “Pulmonary Embolism.” The following list outlines where to view guests and topics:

**Video 1:** The editors and authors discuss details and explore overarching themes in Issue 20.3, titled “Pulmonary Embolism.”

**WELCOME**

Alan Lumsden, MD, introduces topic and co-guest editor (2:07)

Thomas Loh, MD, offers overview of theme and topics (3:15)

**“NEW DIAGNOSTIC TOOLS FOR PULMONARY EMBOLISM DETECTION”**

Dr. Loh introduces Patrick Muck, MD (6:30)

Dr. Muck begins (8:08)

**INTRODUCTION OF PANEL**

Dr. Loh introduces Pavan Thangudu, MD (12:18)

Dr. Loh introduces Joseph Naoum, MD (12:42)

Dr. Loh introduces Nicolas Mouawad, MD (12:57)

Dr. Loh introduces Paul Haddad, MD (13:09)

Dr. Loh introduces Zachary Steinberg, MD (13:22)

Dr. Loh introduces Paul Haddad, MD (30:00)

**CONVERSATION: ESTABLISHING PROTOCOLS & STANDARDIZATION OF CARE FOR PULMONARY EMBOLISM AND ST-SEGMENT ELEVATION MYOCARDIAL INFARCTION (STEMI)**

Dr. Lumsden poses question (13:35)

Dr. Steinberg responds (14:38)

**“FROM TRENDELENBURG TO PERTS: EVOLUTION IN THE MANAGEMENT OF MASSIVE PULMONARY EMBOLISM”**

Dr. Loh poses question for Pavan Thangudu, MD (16:00)

Dr. Thangudu begins (17:08)

**CONVERSATION: “ANTICOAGULATION MANAGEMENT POST PULMONARY EMBOLISM”**

Dr. Loh poses question (23:22)

Dr. Loh poses question for Nicolas Mouawad, MD (24:02)

Dr. Mouawad begins (24:37)

Dr. Muck contributes on artificial intelligence (28:05)

Dr. Lumsden contributes on costs/compliance (29:00)

**“CATHETER INTERVENTIONS FOR PULMONARY EMBOLISM: MECHANICAL THROMBECTOMY VERSUS THROMBOLYTICS”**

Dr. Loh poses question for Nicolas Mouawad, MD (30:24)

Dr. Mouawad begins (31:10)

Dr. Loh poses question for Joseph Naoum, MD (33:20)

Dr. Naoum begins (33:36)

Dr. Loh poses question for Paul Haddad, MD (35:42)

Dr. Haddad begins (36:00)

**CONVERSATION: EDUCATION & TRAINING FOR PULMONARY EMBOLISM**

Dr. Loh poses question for Paul Haddad, MD (36:37)

Dr. Haddad begins (37:11)

Dr. Loh asks guest panel (37:57)

Dr. Mouawad begins (38:26)

Dr. Thangudu responds (39:57)

Dr. Steinberg responds (42:45)

Dr. Lumsden responds (45:20)

Dr. Naoum responds (47:03)

Dr. Lumsden responds (48:32)

**CONVERSATION: WHAT’S THE MECHANISM FOR OPENING UP FLOW?**

Dr. Lumsden poses question (48:55)

Dr. Loh responds (49:17)

**“INFERIOR VENA CAVA FILTERS: AN OVERVIEW”**

Dr. Loh poses question (50:15)

Dr. Haddad begins (51:03)

Dr. Loh asks guest panel (51:54)

Dr. Naoum responds (52:21)

Dr. Muck responds (53:28)

Dr. Lumsden responds (53:55)

Dr. Loh poses question (55:32)

Dr. Steinberg responds (56:13)

Dr. Thangudu responds (1:00:19)

## Conclusion

Dr. Lumsden wrap-up (1:00:15)

